# Genetics and biological characteristics of duck reoviruses isolated from ducks and geese in China

**DOI:** 10.1186/s13567-025-01470-7

**Published:** 2025-02-06

**Authors:** Xiaohong Sun, Jing Guo, Jinyan Shen, Mengdi Guan, Lili Liu, Yujiao Xie, Hongke Xu, Mengjing Wang, Anran Ren, Wenxi Li, Feng Cong, Xuyong Li

**Affiliations:** 1 College of Agriculture and Biology, Liaocheng University, Liaocheng, China; 2 Guangdong Laboratory Animals Monitoring Institute, Guangzhou, China

**Keywords:** Duck reovirus (DRV), avian orthoreovirus (ARV), evolution, pathogenicity, duck, goose, chicken

## Abstract

The emergence and circulation of duck reovirus have caused severe threats to domestic waterfowl production because of the lethal infections they cause in ducks and geese. However, the evolution of circulating duck reoviruses and their replication and pathogenicity in domestic birds have not been fully investigated. In this study, we identified and isolated six duck reoviruses from clinical samples of sick or deceased farmed ducks and geese and sequenced their full genomes. Phylogenetic analysis revealed the evolutionary landscape of duck reoviruses and the complex reassortment of these circulating viruses with avian orthoreovirus and Muscovy duck reovirus. Animal infection studies revealed differences in the replication and pathogenicity of the reoviruses identified in this study in ducks, geese and chickens. Lethal infection with highly pathogenic viruses causes severe focal necrosis and hemorrhage in the liver, spleen, bursa of Fabricius and thymus, resulting in high mortality in inoculated birds. Importantly, chickens are susceptible to circulating duck reovirus, highlighting the potential risk of duck reovirus infection in chickens. Our study revealed the evolution, pathogenicity and potential cross-species transmission risk of duck reoviruses, further emphasizing the importance of continued and systemic surveillance at the interface of domestic waterfowl and chickens.

**Supplementary Information:**

The online version contains supplementary material available at 10.1186/s13567-025-01470-7.

## Introduction

Avian reoviruses (ARVs) are members of the genus *Orthoreovirus* in the family *Reoviridae* and contain ten segmented double-stranded RNAs (dsRNAs) within a nonenveloped icosahedral double-capsid shell [1, 2]. The genomes of ARVs are divided into four small segments (S1, S2, S3, and S4), three medium segments (M1, M2, and M3), and three large segments (L1, L2, and L3) according to the length of the ten RNA segments. At least twelve proteins, σ (σA, σB, σC, σNS), P (P10, P18), μ (μA, μB, μNS) and λ (λA, λB, λC), are encoded by the ten segments [3, 4]. The proteins σB and σC, which are encoded by the S3 and S1 genes, are considered the most important structural proteins for determining the antigenicity of ARVs [5–7].

ARVs can infect a wide range of hosts, including wild birds and domestic birds, leading to significant economic losses in the poultry industry. To date, reoviruses have been identified in chickens [8, 9], turkeys [10], domestic ducks [11], geese [12], pigeons [13], mallards [14] and wild birds [15–17]. Generally, orthoreoviruses in birds are classified as chicken reovirus (commonly named ARV), Muscovy duck reovirus (MDRV) (classic waterfowl-origin), or duck reovirus (commonly named novel duck reovirus; DRV or NDRV) according to their genetic differences, host reservoirs, and pathogenicity in birds. ARV infections in broilers and other domestic birds have been detected globally for more than half a century [18–20]. MDRVs are mainly found in Muscovy ducks, resulting in necrotic liver foci and high mortality in Muscovy ducklings [21].

In the past two decades, DRV (NDRV) has emerged in Southeast China and has circulated in a variety of duck species, causing severe hemorrhagic and necrotic lesions in the liver and spleen of infected ducks [22–24]. More importantly, DRV has been introduced in geese, and similar clinical symptoms, such as hemorrhagic and necrotic lesions in the liver and spleen, have been observed in geese [12, 25, 26]. Zhang et al. reported a novel reovirus isolated from geese and reported that the virus was a reassortant of DRV, MDRV and chicken orthoreovirus [27]. Our previous study reported that DRVs were detected in the fecal samples of wild ducks, egrets, and swans. Phylogenetic analysis revealed that these wild bird-originating viruses are closely related to the circulating duck viruses in China [16]. Despite continuous reports on the circulation of DRV in ducks and geese, the genetic and evolutionary landscape of DRV and its potential differential pathogenicity in ducks, geese and chickens are largely unknown.

In this study, we isolated six DRVs from clinical samples of ducks and geese in 2022 and sequenced their full genomes. The detailed genetic and phylogenetic analysis of the available DRV strains will help us understand the evolutionary trends of these viruses. The findings of the experimental infection studies in ducks, geese and chickens further revealed the variety of replication and virulence phenotypes of the circulating viruses. These findings help fill the knowledge gap concerning DRVs and contribute to viral surveillance in domestic birds.

## Materials and methods

### Experimental animals

One-day-old specific pathogen-free (SPF) chickens and one-day-old SPF ducks were purchased from Shandong Healthtech Laboratory Animal Breeding Co., Ltd. (Ji’nan, Shandong, China). One-day-old goslings were purchased from a local hatchery and were tested to exclude potential infection with reovirus, astrovirus, avian influenza virus, parvovirus, or gosling plague virus. The grouped birds were housed in biosafety isolators during the experimental period.

### Viruses and cells

The six reoviruses used in this study were isolated from clinical samples from ducks and geese in Shandong Province, China, in 2022. Leghorn male hepatoma (LMH) cells were cultured with DMEM containing 10% fetal bovine serum (FBS) at 37 °C. The egg median lethal dose (ELD_50_) of the tested viruses was measured in 9–10-day-old chicken embryos.

### Sample collection

Samples were collected from sick or dead ducks and geese on local farms in Shandong Province in 2022. The samples (liver, spleen, heart, kidney, pancreas, and lung) were stored in a −80 °C freezer for viral identification and isolation.

### Virus identification and isolation

The frozen samples were homogenized and centrifuged at 12 000 rpm for five minutes, and the supernatant was then filtered through 0.22 μm filters. The RNA of the supernatant samples was extracted via a DNA/RNA extraction kit and then identified via RT‒PCR via specific viral primers. The specific viral primers used targeted duck Tembusu virus (DTMUV), avian influenza virus (AIV), Newcastle disease virus (NDV), duck hepatitis virus-1 (DHV-1), duck hepatitis virus-3 (DHV-3), fowl adenovirus (FAdV), goose astrovirus (GAstV), and duck reovirus (DRV-σC), which are described in Additional file 1. The filtered samples positive for DRV were then inoculated into 9-day-old SPF chicken embryos and LMH cells for virus isolation. The isolated viruses were then purified by limiting serial dilution method and amplified by two passages in both chicken eggs and LMH cells. The egg median lethal dose (ELD_50_) of the purified viruses was then tested in 9-day-old chicken egg embryos. The reoviruses isolated from chicken embryos and LMH cells were stored at −80 °C for further use.

### Electron microscopy

The reovirus was inoculated into LMH cells for 96 h, and the culture supernatant was pelleted by ultracentrifugation at 30 000 rpm for 3 h. The pellets were then resuspended in TNE buffer and purified by sucrose density gradient centrifugation at 28 000 rpm for 2 h. The virus band was collected and pelleted by ultracentrifugation again. The obtained virions were stained with phosphotungstic acid according to standard procedures and observed via transmission electron microscopy (TEM).

### Indirect immunofluorescence assay (IFA)

The LMH cells were cultured in a 6-well plate, and the reoviruses were inoculated onto the cells. One hour later, the cells were washed three times with phosphate-buffered saline (PBS) and then cultured at 37 °C in a 5% CO_2_ incubator for 8 h. The supernatants of the wells were discarded, and the cells were washed three times with PBS and then fixed with 4% paraformaldehyde at room temperature for 10 min. The fixed cells were incubated with 0.5% Triton X-100 in PBS for 30 min and blocked with 1% bovine serum albumin (BSA) at room temperature for 30 min. A mouse-derived polyclonal primary antibody and a goat anti-mouse IgG secondary antibody (TransGen Biotech, HS211-01) against the DRV σC protein were used to detect the viruses. After incubation with 1 μg/mL DAPI (Beyotime, China), the cells were washed and examined via a fluorescence microscope.

### Genome sequencing of reoviruses

The viral RNA of the identified viruses was extracted from the allantoic fluid of virus-infected eggs or LMH cell supernatant, and cDNA was synthesized via reverse transcription (RT) via random primers. The PCR products amplified by the gene-specific primers (Additional file 2) were sequenced on an Applied Biosystems DNA Analyser (3500xL Genetic Analyser, USA) according to the manufacturer’s instructions. The full-length sequences of the viral genomes were edited and assembled via the SeqMan program (DNASTAR, Madison, WI, USA).

### Phylogenetic analysis

Phylogenetic analyses were performed on the basis of the sequences of the six viruses investigated in this study and the available sequences of reoviruses downloaded from GenBank. Multiple sequence alignment was performed via MAFFT software (v7.505), and the sequences were then trimmed via MEGA11. The sequences of the six duck reoviruses were compared via MegAlign in the Lasergene (v11.0) package for calculation of similarity. A maximum clade credibility (MCC) phylogenetic tree was constructed on the basis of the S3 gene of reoviruses and nucleotide sequences encoding the protein σC via BEAST (v1.10.4). The optimal nucleotide substitution model was determined via IQ-tree (v2.2.0). The GTR + F + G4 model was selected as the best substitution model, with a lognormal uncorrelated relaxed clock as the clock model. Parameters with effective sample sizes greater than 200 were accepted. The MCC tree was generated for each dataset via Tree Annotator in BEAST after a burn-in of 10%. For the S1, S2, S3, S4, M1, M2, M3, L1, L2 and L3 genes, a maximum likelihood (ML) phylogenetic tree was constructed via IQ-TREE with 1000 ultrafast bootstrap replicates. The tvBOT online service was used to visualize the trees.

On the basis of the neucleotide homology and phylogenetic analysis, the most related sequences of each gene segment of the six viruses were further analysed. The viral information of the most related sequences, including the host, isolation region and time of the viruses, was retrieved from GenBank to support reassortment events between chicken viruses and duck viruses. The reassortment event was identified if the duck viruses clustered together with the chicken viruses in the phylogenetic tree and shared high genetic nucleotide identity with the circulated chicken orthoreovirus.

### Duck study

Seventy one-day-old SPF ducklings were divided into seven groups. The grouped ducks were inoculated with the indicated virus by leg muscle injection with a volume of 200 µL at a concentration of 10^4^ ELD_50_/mL (100 µL/leg). Ten ducks inoculated with PBS were used as the negative control. Three birds from each group were euthanized at 3 days post-infection (dpi), and the indicated samples (thymus, liver, spleen, bursa of Fabricius, brain, heart, lung, intestine, and kidney) were collected for viral titration in eggs. Viral titres were calculated via a chicken embryo inoculation (ELD_50_) assay. Two birds from each inoculated group were euthanized at 3 dpi, and samples (thymus, liver, spleen, and bursa of Fabricius) were collected for histological analysis. The remaining five ducks were monitored daily for 14 days for weight loss and survival.

### Goose study

Thirty one-day-old geese were purchased from a local farm. Oropharyngeal swabs and cloacal swabs were collected from the geese to ensure that the goslings were negative for DTMUV, AIV, NDV, GAstV, and DRV by PCR. Thirty geese were divided into three groups. The pathogenicity of HZ01/2022 and JN06/2022 was tested in geese. The methods of virus inoculation, sample collection, virus titration and histological studies were the same as those used for the duck study, as described above.

### Chicken study

Seventy one-day-old SPF chickens were divided into seven groups. The methods were the same as those used for the duck and goose studies, as described above.

### Histopathology analysis

The liver, spleen, bursa of Fabricius, and thymus of the ducks, geese and chickens were harvested at 3 dpi and fixed in 10% neutral-buffered formalin solution. The fixed samples were embedded in paraffin and then sliced into 4 µm sections for hematoxylin–eosin (H&E) staining. The tissue slides were examined for lesions via light microscopy.

## Results

### Identification of the DRV from clinical samples

**Figure 1:**
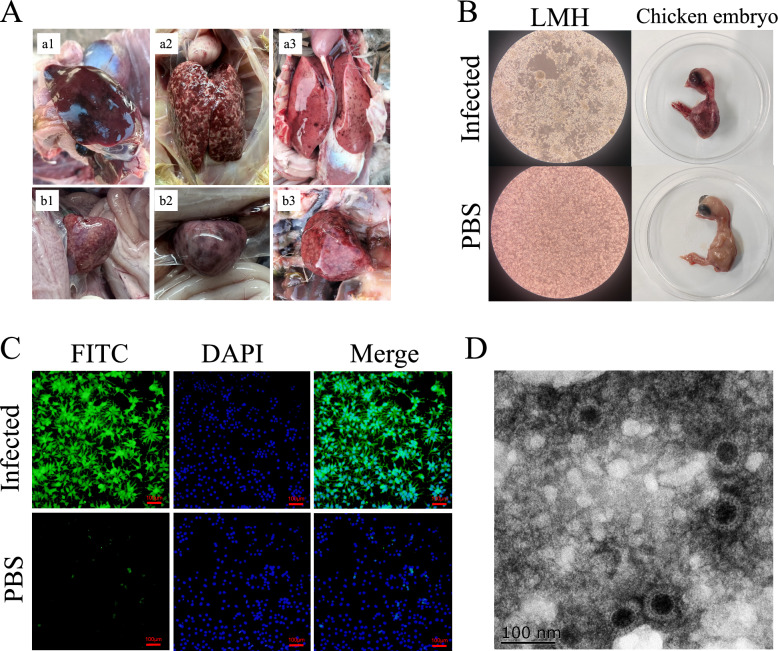
**Identification and isolation of duck reoviruses from clinical duck and goose samples**. **A** Lesions in the liver (a1, a2, a3) and spleen (b1, b2, b3) of dead ducks (a1, b1) and geese (a2, b2, a3, b3) collected from poultry farms. **B** Typical lesions in LMH cells and chicken embryos caused by duck reoviruses. The PCR-positive clinical samples were inoculated with LMH cells or 9–10-day-old chicken embryo eggs to isolate the duck reoviruses. **C** Identification of duck reoviruses in LMH cells via indirect immunofluorescence assay (IFA). The duck reoviruses were inoculated into LMH cells, and infection was confirmed by IFA using an anti-σC antibody. **D** Viral morphogenesis observed via electron microscopy. The viruses grown in LMH cells were concentrated, negatively stained, and examined via transmission electron microscopy (TEM).

Sick/dead ducklings or goslings were sampled from duck and goose farms in Shandong Province in 2022. Severe hemorrhage and necrosis of the liver and spleen were detected in some of the ducks (Figure 1A, panels a1, b1) and geese (Figure 1A, panels a2, b2, a3, b3). The collected liver and spleen samples were first subjected to RT‒PCR with specific primers for the identification of viruses commonly found in ducks and geese, including DRV, MDRV, duck hepatitis A virus (types 1 and 3), GAstV (type 2), AIV, DTMUV and duck parvovirus (Additional file 1). Chicken embryos and chicken-derived cell lines have been widely used to isolate DRV from samples [20, 28, 29]. In this study, DRV-positive samples were inoculated into SPF chicken embryos and LMH cell cultures for virus identification and isolation, respectively. The inoculated chicken embryos were killed within 72 h, and embryonic hemorrhage and developmental retardation were detected in these inoculated eggs (Figure 1B). The DRV from the embryonic mixture was then confirmed by RT‒PCR (data not shown), suggesting that DRV can replicate in chicken embryos but is lethal to eggs. The LMH cells were also inoculated with the DRV-positive samples and showed a cytopathic effect (CPE) within 72 h (Figure 1B). The supernatants were tested via RT‒PCR (data not shown), and the viruses in the inoculated cells were confirmed via indirect immunofluorescence assay (IFA) via a mouse-derived anti-sigma C (σ C) antibody (Figure 1C). Additionally, DRV virions were detected by TEM in LMH cell culture (Figure 1D). In this study, we successfully identified and isolated six DRVs from three duck clinical samples and three goose clinical samples collected ≈chicken eggs were further used in this study.

### Phylogenetic analysis of DRV

**Figure 2:**
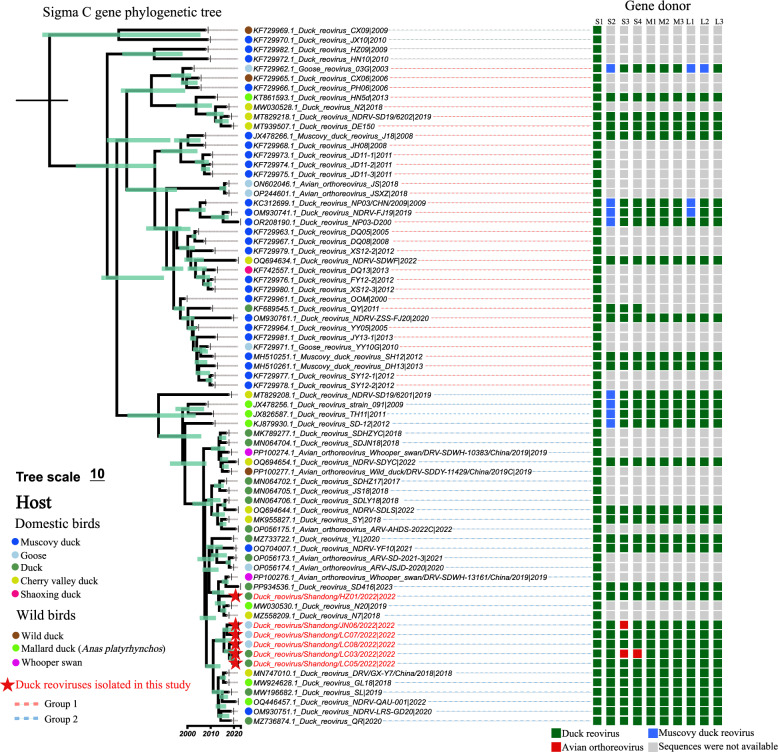
**Phylogenetic analysis of sigma C (σC) nucleotide sequences of the duck reoviruses, including the six duck viruses in this study** **(*n* = 73 sequences).** The sigma C gene sequences of the duck reoviruses were retrieved from the GenBank database (*n* = 67) to construct a time-scaled tree with the viruses detected in this study. The fragment size of the σC nucleotide sequences is 966 nt (571–1536) of S1 gene (1568 nt). The gene donors of each gene segment of the reoviruses included in the phylogenetic tree were further classified to reveal the reassortment events.

To understand the genomic characteristics of the viruses, the whole genomes of the six reoviruses were sequenced and deposited in GenBank (Table 1). We then performed phylogenetic analysis of each gene segment to reveal the evolution of the reoviruses. The σ C protein is one of the most important nucleocapsid proteins and is encoded by ORF 3 of the reovirus S1 segment. The time-scaled phylogenetic tree of the reovirus σ C gene was inferred from the σ C nucleotide sequences of DRV, including the six viruses sequenced in this study and 73 σ C nucleotide sequences of DRV available from GenBank, according to their collection time and host species (Figure 2). Overall, the σ C genes of the recently circulating DRVs were clustered into two main groups (Group 1 and Group 2). The Group 1 viruses were mostly found in Muscovy ducks, whereas the Group 2 viruses were commonly detected in both domestic ducks and geese. Moreover, Group 2 viruses were detected in wild birds, including mallards, swans and wild ducks, suggesting their expanded host range. The six viruses detected in this study clustered into Group 2 and shared high genetic similarity with the viruses recently found in ducks in China (Figure 2). The σ C genes of the six viruses isolated from ducks and geese in this study shared 97.9–99.7% nucleotide similarity, suggesting that the σ C genes of the three DRVs isolated from geese originated from viruses that circulated in ducks.

**Table 1:** **Information on the duck reoviruses isolated in this study**

No	Viruses	Abbreviation	Sample information			Passage history	GenBank Accession Number
			Collected date	Location	Species		
1	Duck reovirus/Shandong/HZ01/2022	HZ01/2022	March, 3, 2022	Heze, Shandong	Duck	Chicken egg embryos, passage 2	PQ330126-PQ330135
2	Duck reovirus/Shandong/LC03/2022	LC03/2022	March, 10, 2022	Liaocheng, Shandong	Duck		PQ330136-PQ330141 PQ346590-PQ346593
3	Duck reovirus/Shandong/LC05/2022	LC05/2022	March, 10, 2022	Liaocheng, Shandong	Duck		PQ346594-PQ346603
4	Duck reovirus/Shandong/JN06/2022	JN06/2022	February, 23, 2022	Jining, Shandong	Goose		PQ346604- PQ346613
5	Duck reovirus/Shandong/LC07/2022	LC07/2022	February, 10, 2022	Liaocheng, Shandong	Goose		PQ346614- PQ346623
6	Duck reovirus/Shandong/LC08/2022	LC08/2022	February, 27, 2022	Liaocheng, Shandong	Goose		PQ346624- PQ346633

**Figure 3:**
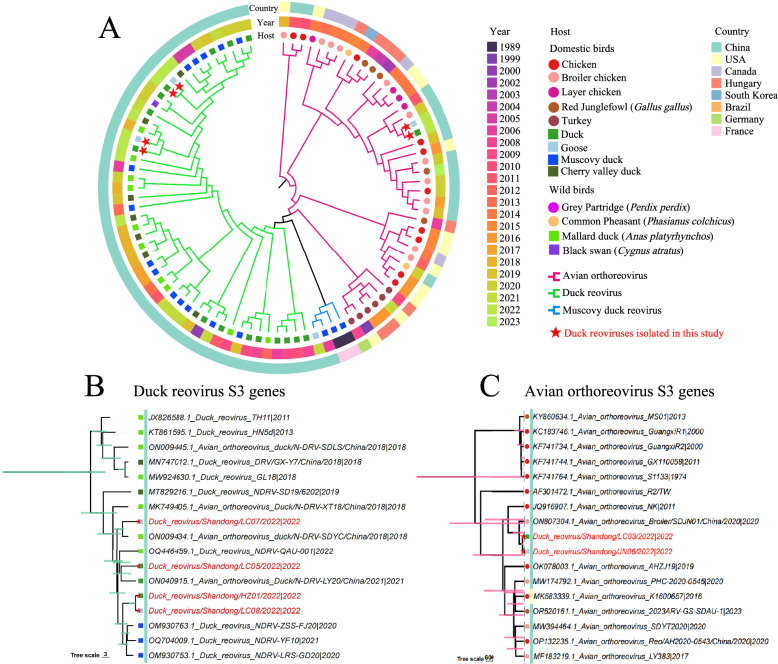
**Phylogenetic analysis of S3 gene sequences of avian and waterfowl reoviruses**. **A** Phylogenetic tree of the S3 genes of the reoviruses detected in different avian hosts and regions of the world, including six sequences of duck reoviruses detected in this study in 2022 (*n* = 94 sequences in total). **B** Phylogenetic tree of the S3 genes of duck reoviruses (*n* = 17). **C** Phylogenetic tree of the S3 genes of avian orthoreoviruses (*n* = 17). The sequences highlighted in red in panels B and C are the viruses sequenced in this study. The fragment size of the S3 gene is 1104 nt (31–1134).

σ B is another important nucleocapsid protein that is encoded by the reovirus S3 gene segment. To reveal the genetic and phylogenetic relationships of the S3 gene of avian (avian, duck, and Muscovy duck) reoviruses, we retrieved the available S3 nucleotide sequences from GenBank to construct a phylogenetic tree. The S3 genes of avian reoviruses generally cluster into three sublineages: the ARV sublineage, the MDRV sublineage, and the DRV (NDRV) sublineage. The ARV sublineage is found mainly in chickens globally and can be considered an ancestor of the descendant MDRV and DRV sublineages (Figure 3A). The viruses of the DRV sublineage were detected mainly in ducks, Muscovy ducks and geese in China. The S3 genes of four viruses isolated from ducks (HZ01/2022 and LC05/2022) and geese (LC07/2022 and LC08/2022) clustered in the DRV sublineage, with high nucleotide identity with the recent viruses detected in ducks (Figure 3B). Interestingly, the S3 genes of two DRVs (LC03/2022 and JN06/2022, which were isolated from ducks and geese, respectively) clustered into the ARV sublineage, which has not been previously reported (Figure 3C). Phylogenetic analysis revealed that the S3 genes of LC03/2022 and JN06/2022 are very closely related to those of the chicken-derived viruses Broiler/SDJN01/China/2020 detected in Shandong Province (neucleotide identities of 99% and 99.17%, respectively). These results suggested that the S3 segments of LC03/2022 and JN06/2022 originated from circulating ARVs in chickens.

The S1, S2, S3, S4, M1, M2, M3, L1, L2, and L3 segments of the six reoviruses identified in this study shared 98 to 99.7%, 96.6 to 100%, 67.6 to 99.8%, 78.6 to 100%, 99.3 to 100%, 97.9 to 99.8%, 99 to 100%, 98.1 to 99.9%, 98.3 to 99.9%, and 98.8 to 99.9% similarity at the nucleotide level, respectively (Additional file 3). Except for the S3 and S4 segments, the other eight gene segments of the six viruses shared high genetic similarity with the duck-derived viruses and clustered in the DRV sublineage (Additional file 4). Notably, the S4 gene segment of LC03/2022 clustered into the ARV sublineage and shared high genetic identity (100% similarity at the nucleotide level) with the chicken-derived virus GX/2010/1 detected in China [30], suggesting that the S4 gene of this virus originated from ARVs circulating in chickens (Additional file 4).

On the basis of the available DRV sequences in GenBank and their phylogenetic diversity, we attempted to determine the genome constellation of these identified viruses. Although most of the detected viruses were partially sequenced (39 of 73 viruses were not fully sequenced), gene segment reassortment between DRV and MDRV was observed in the previously identified viruses. Importantly, two DRV and ARV reassortants were first identified from clinical duck and goose samples in this study (Figure 2). These genetic analysis results indicate that DRVs have undergone continued evolution and complicated reassortment in waterfowl.

### Replication and virulence of DRV in ducks

**Figure 4:**
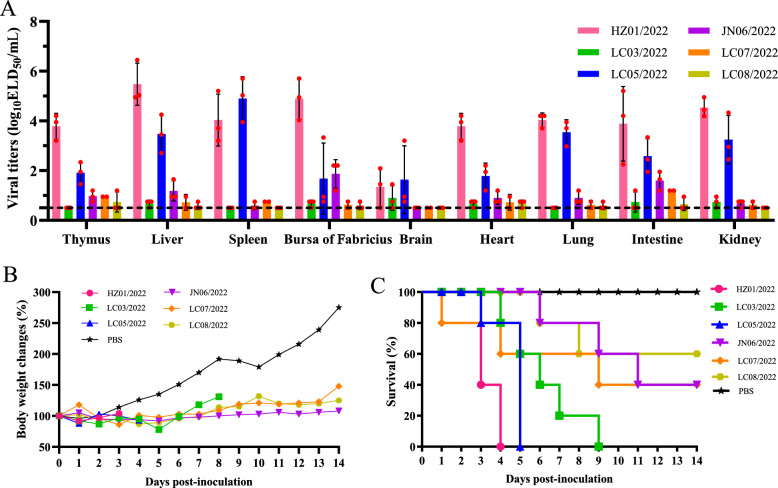
**Replication and virulence of duck reoviruses in SPF ducklings**. **A** Replication of the six viruses in SPF ducks. The dashed line indicates the lower detection limit. **B** Body weight changes in ducklings inoculated with the reoviruses. **C** Survival of ducklings inoculated with the reoviruses.

To better understand the differences in replication and virulence of DRV in ducks, SPF ducks were inoculated with the six viruses detected in this study. One-day-old ducks were inoculated through leg muscle injection with the tested viruses, and organ and tissue samples were collected at 3 dpi for viral titration in chicken eggs. Importantly, the six viruses presented different replication abilities in ducks. The two duck-originating viruses, HZ01/2022 and LC05/2022, replicated in all the collected tissue samples with high viral titres (ranging from 1.45 to 5.7 log_10_ ELD_50_/mL); however, another duck-originating virus (LC03/2022) and the three goose-originating viruses replicated in some of the collected organs, with limited viral titres (Figure 4A). Compared with the ducks inoculated with PBS, all the ducks inoculated with the reovirus presented clinical signs of depression-like symptoms and growth retardation during the observation period (Figure 4B). Signs of lethargy, anorexia and severe diarrhoea were also observed in the inoculated ducks during the observation period (data not shown). The viruses HZ01/2022 and LC05/2022 killed all five birds within five days, suggesting their high pathogenicity in ducklings (Figure 4C). Interestingly, despite the limited replication ability in ducks, the other four viruses presented moderate-to-high pathogenicity to ducks because 40% to 100% of inoculated ducks died during the observation period (Figure 4C).

**Figure 5:**
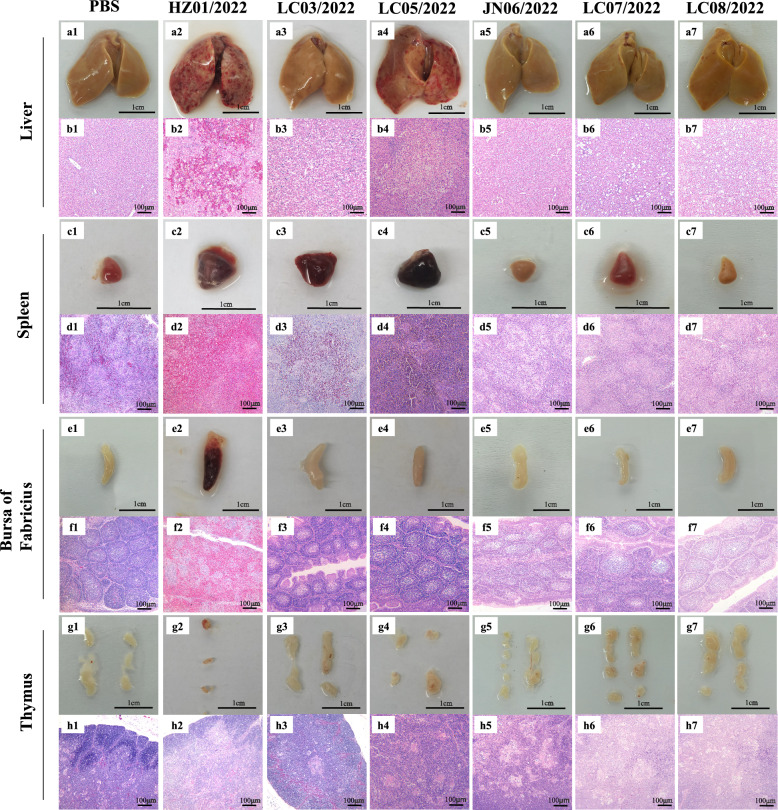
**Pathological lesions of ducks**. Ducks were inoculated with the indicated viruses, and the liver, spleen, bursa of Fabricius and thymus were collected for imaging and HE staining at 3 dpi. The samples were fixed at the same position for imaging, and the HE-stained sections were magnified 100 times.

To further investigate the virulence of DRVs, we then performed pathological studies on the organs of the inoculated ducks. The liver, spleen, bursa of Fabricius, and thymus of the ducks were collected to observe pathological lesions. Various degrees of lesion severity in the sampled organs caused by the six tested viruses were observed. Clinical pathological changes, such as hemorrhagic spots, hemorrhagic points, necrotic foci, and atrophy, were observed in the livers of these inoculated ducks. Compared with the other interventions, the HZ01/2022 and LC05/2022 viruses presented greater pathogenicity because dense hemorrhagic spots, numerous foci of necrosis, extensive hepatocellular degeneration and necrosis were detected in the livers of the ducks (Figure 5, Panels a1-a7). Hepatocellular degeneration, necrosis, and lymphocytic infiltration were further observed via HE staining microscopy (Figure 5, panels b1-b7). With three duck-originated viruses (HZ01/2022, LC03/2022 and LC05/2022), splenomegaly, hemorrhage, and destruction of intrinsic tissues of the spleen were observed, whereas structural disorders of the spleen without splenomegaly and hemorrhage were observed with the three goose-originating viruses (Figure 5, panels c1-c7, d1-d7). Notably, HZ01/2022 induced bleeding and redness of the bursa of Fabricius, whereas LC07/2022 induced atrophy of the bursa of Fabricius (Figure 5, panels e1-e7, f1-f7). Thymus atrophy was also observed in the HZ01/2022 group, while organomegaly was found in the LC03/2022, LC05/2022, LC07/2022 and LC08/2022 groups (Figure 5, panels g1-g7, h1-h7). In summary, duck- and goose-originated reoviruses exhibit various replication abilities and pathogenicities in ducklings.

### Replication and virulence of DRV in geese

**Figure 6:**
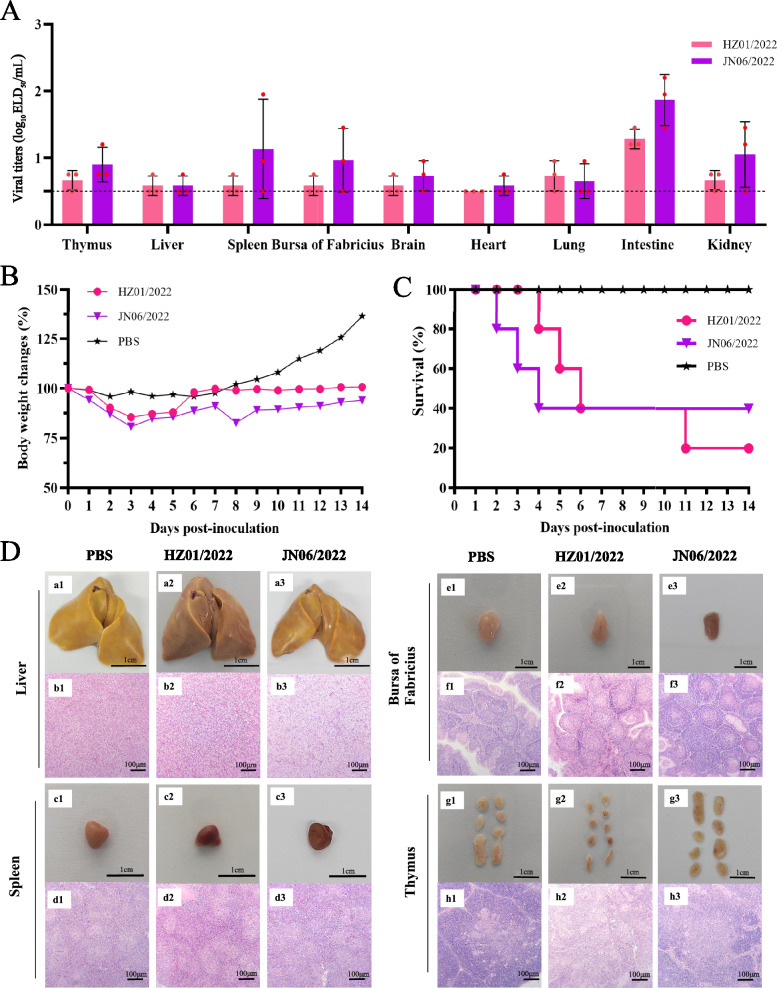
**Replication and virulence of duck reoviruses in geese**. **A** Replication of the two tested viruses in geese. The dashed line indicates the lower detection limit. **B** Body weight changes of goslings inoculated with the reoviruses. **C** Survival of goslings inoculated with the reoviruses. **D** Pathological lesions in the liver, spleen, bursa of Fabricius and thymus of goslings caused by reovirus infection.

Because three reoviruses in this study were isolated from clinical samples from geese and the infection and virulence of the reoviruses in geese are largely unknown, we then evaluated the incursion risk of the reoviruses in goslings. One virus, HZ01/2022, which exhibited high virulence in ducks, and another goose-originating reassortant, JN06/2022, were used to test their replication and pathogenicity in geese. One-day-old goslings were inoculated through leg muscle injections with the tested viruses, and the organs and tissues were collected at 5 dpi for virus titration. The HZ01/2022 virus was detected in all organs and tissues except for the heart, with viral titres ranging from 0.75 to 1.45 log_10_ ELD_50_/mL. The JN06/2022 virus was detected in all the samples from the inoculated geese, with viral titres ranging from 0.75 to 2.2 log_10_ ELD_50_/mL (Figure 6A). The two tested viruses induced up to 14.43% and 19.25% body weight loss in the inoculated geese, and growth retardation was also found in both virus-inoculated groups compared with the PBS-inoculated group (Figure 6B). Clinical signs, such as anorexia, severe diarrhoea, and growth retardation, can be found in inoculated goslings. Importantly, both viruses were lethal to the inoculated goslings, as 80% and 60% of the geese died during the observation period, respectively (Figure 6C). Significant pathological lesions, including bleeding of the spleen and bursa of Fabricius, were observed in the virus-inoculated groups; however, necrotic foci or spots in the liver or spleen of the infected goslings were not observed. Pathological lesions, such as those associated with cellular degeneration, lymphocytic infiltration and structural disorders, were detected via HE staining (Figure 6D). These results indicate that reoviruses isolated from ducks and geese can replicate in geese and are highly pathogenic to goslings.

### Replication and virulence of DRV in chickens

**Figure 7:**
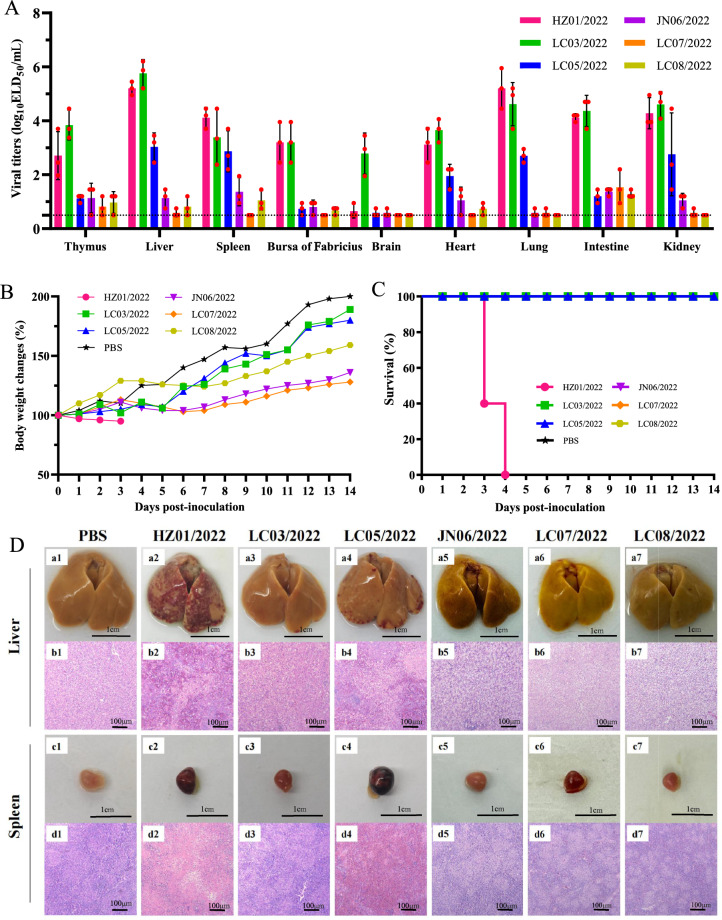
**Replication and virulence of duck reoviruses in SPF chickens**. **A** Replication of the six viruses in SPF chickens. The dashed line indicates the lower detection limit. **B** Body weight changes of the chickens inoculated with the reoviruses. **C** Survival of chickens inoculated with the reoviruses. **D** Pathological lesions in the liver and spleen of chickens caused by reoviruses.

The reassortants detected in this study indicate that cocirculation of ARV, MDRV, and DRV at the interface of terrestrial birds and waterfowl may contribute to the emergence of reassorted reoviruses. To date, DRVs have not been isolated from chickens, and the risk of DRV infection in chickens is largely unknown. We further evaluated the infection potential and virulence of the six reoviruses in chickens. One-day-old SPF chickens were inoculated with the viruses via leg muscle injection, and the organs and tissues were collected for virus titration and pathological studies. Surprisingly, duck-originating reoviruses, especially HZ01/2022 and LC03/2022, were detected in all the collected samples and efficiently replicated, with viral titres ranging from 0.95 to 6.2 log_10_ ELD_50_/mL. The three goose-originating reoviruses were detected in most of the collected samples, with relatively low viral titres (Figure 7A). The clinical signs of the inoculated chickens, including depression, anorexia, severe diarrhoea, and growth retardation, were found during the observation period. The growth retardation of the chickens in the six virus-inoculated groups was greater than that in the PBS-inoculated group (Figure 7B). Interestingly, the HZ01/2022 virus exhibited high pathogenicity in chickens, as 100% of the chickens died within four dpi (Figure 7C). No deaths were observed in the other five virus-inoculated groups within two weeks post-inoculation (Figure 7C).

Pathological studies were then performed to reveal the virulence of these reoviruses in chickens. Massive necrotic foci and hemorrhagic spots were observed in the livers of infected chickens in the HZ01/2022 and LC05/2022 groups, and multiple necrotic and hemorrhagic foci in the liver were detected by microscopic examination, which was similar to the pathological changes in ducks. Small bleeding spots on the surface of the liver, as well as hepatocellular steatosis, were observed in the other four groups (Figure 7D, panels a1-a7, b1-b7). Another important lesion caused by the viruses (HZ01/2022 and LC05/2022) is bleeding and swelling of the spleen, as extensive and severe red blood cell accumulation and destruction of intrinsic tissues were detected via HE staining (Figure 7D, panels c1-c7, d1-d7). Pathological lesions in the bursa of Fabricius, such as lymphoid follicle atrophy and decreased lymphocyte count, were detected in the infected chickens (Additional file 5, Panels a1-a7, b1-b7). Swelling (LC03/2022, LC05/2022), atrophy (JN06/2022, LC07/2022, LC08/2022), or haemorrhage (LC07/2022) were detected in the thymus of chickens. Microscopy revealed structural disorganization, glandular atrophy, and a decrease in the number of lymphocytes in the thymus of the inoculated chickens (Additional file 5, panels c1-c7, d1-d7). These findings indicate that duck- and goose-originating reoviruses present high incursion risks to chickens because of their infectivity and virulence.

## Discussion

In this study, based on clinical samples collected from sick or dead ducks and geese, six duck reoviruses were successfully isolated and fully characterized. Phylogenetic, replication and pathogenicity analyses of the currently circulating DRVs will help us evaluate their threat to poultry production.

DRV has circulated in domestic ducks in China for nearly two decades and has been recognized as an important threat to duck production [31]. Unlike ARV, which mainly circulates in chickens, and MDRV, which is primarily detected in Muscovy ducks, DRV presents a more diverse range of hosts, including domestic ducks, mallards, Muscovy ducks, geese, whopper swans, and wild ducks. Recently, DRV was detected in geese and caused symptoms similar to those in ducks [26, 27, 32, 33]. Our previous study revealed reoviruses in wild ducks and swans that share high genetic similarity with circulating reoviruses in ducks and geese [16], suggesting a potential reservoir for DRV. Continuous surveillance at the interface of different birds is essential for monitoring the incursion of DRV into other domestic birds and wild waterfowl. Compared with duck egg embryos, chicken egg embryos and chicken-derived LMH cells are considered ideal vectors for the isolation of DRV from clinical samples. Our results and those of previous reports confirmed that DRV can be successfully isolated from both chicken embryos and LMH cells [20, 28, 29]. However, there are no reports on whether chicken-derived embryos or LHM cells can promote the adaptation of duck-origin viruses to chickens with neucleotide or amino acid mutations in the genome. A comparison of DRVs isolated from duck embryos and chicken embryos may contribute to understanding genetic adaptation during virus isolation and passage.

Gene segment reassortment is one of the most important mutations of segmented viruses and contributes to the emergence of novel viruses in animals [34]. The cocirculation of DRV, MDRV and ARV at the interface of chickens and domestic waterfowl may collectively prompt the emergence of these novel reassortants in ducks and geese. Phylogenetic analysis of the genome sequences of the available DRVs revealed that DRV continues to evolve in waterfowl and has undergone complicated reassortment with MDRV or ARV. DRV reassortants whose partial gene segments originated from MDRV have been previously identified in ducks and geese [28, 35–37]. In this study, we first reported DRV reassortants in ducks (LC03/2022) and geese (JN06/2022), with the S3 and S4 gene segments originating from the ARV. The surveillance of reoviruses at interfaces, such as poultry farms, live poultry markets and slaughterhouses, should be strengthened to monitor the emergence and circulation of reassortants in chickens and waterfowl.

DRV has been recognized as one of the most threatening pathogens to domestic waterfowl production because of the severe pathological lesions in the liver and spleen caused by DRV infections, leading to high mortality in ducks or geese [26, 38, 39]. In this study, typical clinical symptoms, such as necrosis and hemorrhage in the liver and spleen, were commonly found in samples from sick or dead ducks and geese, suggesting that the liver and spleen are the two major organs targeted by naturally circulating DRVs. The typical lesions in the liver and spleen caused by reoviruses may contribute to clinical diagnosis in the field. Duck studies demonstrated differences in replication and pathogenicity among the six reoviruses, leading to a variety of pathological lesions in the target organs caused by these viruses. Research on geese further revealed that goslings are susceptible to reovirus and that the viruses are lethal in geese. Although DRV infection has not been reported in chickens, the occurrence of gene segment reassortment among DRV, MDRV and ARV increases the risk of DRV infection in chickens. Previous reports have shown that novel DRVs can cause systemic infections and are lethal to chickens. Yu et al. reported that severe pathological lesions, such as focal necrosis and hemorrhage, caused by DRV were present in inoculated chickens [40]. The chicken experiments in this study indicated that the six reoviruses can infect and replicate efficiently in chickens. Notably, the duck-originated HZ01/2022 virus caused severe focal necrosis and hemorrhage in the liver and spleen and led to 100% mortality in the inoculated chickens. These findings suggest that the possibility of cross-species transmission of circulating DRV into chickens should be considered in poultry production.

In conclusion, the results and findings of this study further increase our knowledge of the genetics and biological characteristics of DRVs and highlight their threat to the poultry industry. Comprehensive measures, including active surveillance, effective DRV vaccines, novel antiviral drugs, and enhanced biosecurity facilities, are essential and should be strengthened to prevent DRV infections in waterfowl.

## Supplementary Information

**Additional file 1. The specific primers used for virus identification.**

**Additional file 2. Primers used for segment amplification and genome sequencing.**

**Additional file 3. Sequence homology of the genes (S1, S2, S3, S4, M1, M2, M3, L1, L2, and L3) of the duck reoviruses identified in this study.**

**Additional file 4. Phylogenetic trees of the genes (S1, S2, S3, S4, M1, M2, M3, L1, L2, and L3) of the duck reoviruses**. The full-length sequences of each gene segment of the viruses were first aligned by MEGA 7.0, and a maximum likelihood (ML) phylogenetic tree was constructed via IQ-TREE with 1000 ultrafast bootstrap replicates. S1, 1568 nt; S2, 1251 nt (16-1266); S3, 1104 nt (31-1134); S4, 1104 nt (24-1127); M1, 2199 nt (14-2212); M2, 2028 nt (30-2057); M3, 1908 nt (25-1932); L1, 3882 nt (22-3903); L2, 3780 nt (15-3794); L3, 3858 nt (13-3870).

**Additional file 5. Pathological lesions in the bursa of Fabricius and the thymus of inoculated chickens.**

## Data Availability

The data supporting the conclusions of this article are included within the article and its additional files.
