# Supplementary material for: Genetics and biological characteristics of duck reoviruses isolated from ducks and geese in China
Source: Vet Res. 2025 Feb 6;56:30. doi: 10.1186/s13567-025-01470-7 (PMC11803967; doi:10.1186/s13567-025-01470-7)
Supplement: Supplementary file 1 — Additional file 1. The specific primers used for virus identification. [file 13567_2025_1470_MOESM1_ESM.pdf]

Additional file 1. The specific primers used for virus identification

Primer name	Primer sequence（5’-3’）
DRV-σC	F:ATGGATKGCAACGAGMTGATAC
	R:CTAGCCCGTCGCGACYGT
MDRV-S4	F:GTGGGTAGTTTGCCGCTAGT
	R:TATCGTACGCGGGGTCAATG
DHV-I	F:ACCATGACCYAGCCTTAG
	R:CCAATGTARCTTMCCTTC
DHV-III	F:GATGTAYTTATGGTGCTWAGACGC
	R:ACGAAKCAGCCAATGACG
DPV	F:CAGGAAATGACGTTAGGGGCT
	R:GCATTTCTGCTCGGGCATTT
GAstV-2	F:AGTTTCAACCTCTCCGCCAG
	R:TCACGCAAGCAGTCTGATGT
AIV	F:AGCAAAKGCAGGWG
	R:AGTAGAMACAAGGKTGTTTT
DTMUV	F:GCCAKGGAATTAGGGGTT
	R:TAATCCTCCATMTCAGCCGTGTAG
