# Supplementary material for: Genetics and biological characteristics of duck reoviruses isolated from ducks and geese in China
Source: Vet Res. 2025 Feb 6;56:30. doi: 10.1186/s13567-025-01470-7 (PMC11803967; doi:10.1186/s13567-025-01470-7)
Supplement: Supplementary file 2 — Additional file 2. Primers used for segment amplification and genome sequencing. [file 13567_2025_1470_MOESM2_ESM.pdf]

Additional file 2.The primers used for segment amplification and genome sequencing

Primer name	Primer sequence（5’-3’）
DRV-S1	F:CTAGGCGGTGGTTTTGGTCT
	R:CAACCGGGACCCAAACGATA
DRV-S2	F:AGGAGTCCATGCTTCACAGC
	R:TAATGCGACGAGCGTAACCA
DRV-S3	F:GCGTGTGCCAAACTTTCACT
	R:GGTGGGAGCCTATGTGCATT
ARV-S3	F:ACTACGGAACTTTGCCCTCG
	R:CTTGTTCGTCTTCTGCAGCG
DRV-S4	F:TTCCCGTAACACATCCGTCG
	R:AAGAGTCCAAGTCGTGGCAG
ARV-S4	F:TTTGAGTCCTTGTGCAGCCA
	R:GTAGCGTTTGGTCACCCTCA
DRV-M1	F:ATCGTTCCAGGCTCAAAGGG
	R:AATGGGGTGAAGAGAGCACG
DRV-M2	F:AATGCATCCGATCCCTCGTC
	R:GAAGTCCCTGCTTCCAGGTC
DRV-M3	F:GTGGGGAGACAAACCGATGT
	R:AGGTCAAAGCTCACAGGTCG
DRV-L1	F:AACGCTGCACTGACTAGCTT
	R:ATGGGTCACCATGCCAGAAG
DRV-L2	F:TCATCTCCTCGAATGCTCGC
	R:GGCAGGTCAGTCGTCAAAGA
DRV-L3	F:GACCAGTTCTTGCGTCGGTA
	R:ACCGTATCGACCAGGAGGAA
