# Supplementary material for: Genetics and biological characteristics of duck reoviruses isolated from ducks and geese in China
Source: Vet Res. 2025 Feb 6;56:30. doi: 10.1186/s13567-025-01470-7 (PMC11803967; doi:10.1186/s13567-025-01470-7)
Supplement: Supplementary file 3 — Additional file 3. Sequence homology of the genes (S1, S2, S3, S4, M1, M2, M3, L1, L2, and L3) of the duck reoviruses identified in this study. [file 13567_2025_1470_MOESM3_ESM.pdf]

Additional file 3. Sequence homology of the genes (S1, S2, S3, S4, M1, M2, M3, L1, L2, and L3) of the duck reoviruses identified in this study

	S1					
	HZ01/2022	LC03/2022	LC05/2022	JN06/2022	LC07/2022	LC08/2022
HZ01/2022		98.3	98.2	98.2	98.2	98
LC03/2022			98.9	98.8	98.6	98.8
LC05/2022				99.5	99.4	99.6
JN06/2022					99.7	99.5
LC07/2022						99.6
LC08/2022						

	S2					
	HZ01/2022	LC03/2022	LC05/2022	JN06/2022	LC07/2022	LC08/2022
HZ01/2022		97.3	97.3	97.4	96.6	96.7
LC03/2022			100	99.9	96.9	96.9
LC05/2022				99.9	96.9	96.9
JN06/2022					96.9	97
LC07/2022						99
LC08/2022						

	S3					
	HZ01/2022	LC03/2022	LC05/2022	JN06/2022	LC07/2022	LC08/2022
HZ01/2022		67.6	98.7	67.7	98.3	99.8
LC03/2022			67.6	99.8	67.9	67.6
LC05/2022				67.7	99.3	98.5
JN06/2022					68.1	67.7
LC07/2022						98.2
LC08/2022						

	S4					
	HZ01/2022	LC03/2022	LC05/2022	JN06/2022	LC07/2022	LC08/2022
HZ01/2022		78.9	99.3	99.6	99.6	99.6
LC03/2022			78.6	78.8	78.8	78.8
LC05/2022				99.7	99.7	99.7
JN06/2022					100	100
LC07/2022						100
LC08/2022						

	M1					
	HZ01/2022	LC03/2022	LC05/2022	JN06/2022	LC07/2022	LC08/2022
HZ01/2022		99.3	99.3	99.3	99.3	99.3
LC03/2022			100	100	100	100
LC05/2022				100	100	100
JN06/2022					100	100
LC07/2022						100
LC08/2022						

	M2					
	HZ01/2022	LC03/2022	LC05/2022	JN06/2022	LC07/2022	LC08/2022
HZ01/2022		98.1	98.1	97.9	98	98.8
LC03/2022			99.8	99.5	99.3	99.1
LC05/2022				99.7	99.4	99.2
JN06/2022					99.6	99.1
LC07/2022						99.2
LC08/2022						

	M3					
	HZ01/2022	LC03/2022	LC05/2022	JN06/2022	LC07/2022	LC08/2022
HZ01/2022		99.1	99	99	99.1	99.5
LC03/2022			99.9	99.9	99.9	99.6
LC05/2022				100	99.9	99.5
JN06/2022					99.9	99.5
LC07/2022						99.5
LC08/2022						

	L1					
	HZ01/2022	LC03/2022	LC05/2022	JN06/2022	LC07/2022	LC08/2022
HZ01/2022		98.2	98.1	98.2	98.2	98.1
LC03/2022			99.8	99.9	99.8	99.7
LC05/2022				99.9	99.9	99.7
JN06/2022					99.9	99.7
LC07/2022						99.7
LC08/2022						

	L2					
	HZ01/2022	LC03/2022	LC05/2022	JN06/2022	LC07/2022	LC08/2022
HZ01/2022		98.7	98.7	98.7	98.9	98.3
LC03/2022			99.8	99.8	99.8	98.9
LC05/2022				99.9	99.9	99
JN06/2022					99.8	98.9
LC07/2022						98.9
LC08/2022						

	L3					
	HZ01/2022	LC03/2022	LC05/2022	JN06/2022	LC07/2022	LC08/2022
HZ01/2022		98.8	99.1	99.2	98.8	99.6
LC03/2022			99.6	99.6	99.9	99
LC05/2022				99.9	99.6	99.2
JN06/2022					99.6	99.3
LC07/2022						99
LC08/2022						
