# Supplementary material for: Genetics and biological characteristics of duck reoviruses isolated from ducks and geese in China
Source: Vet Res. 2025 Feb 6;56:30. doi: 10.1186/s13567-025-01470-7 (PMC11803967; doi:10.1186/s13567-025-01470-7)

Additional file 4. Phylogenetic trees of the genes (S1, S2, S3, S4, M1, M2, M3, L1, L2, and L3) of the duck reoviruses

S1 phylogenetic tree

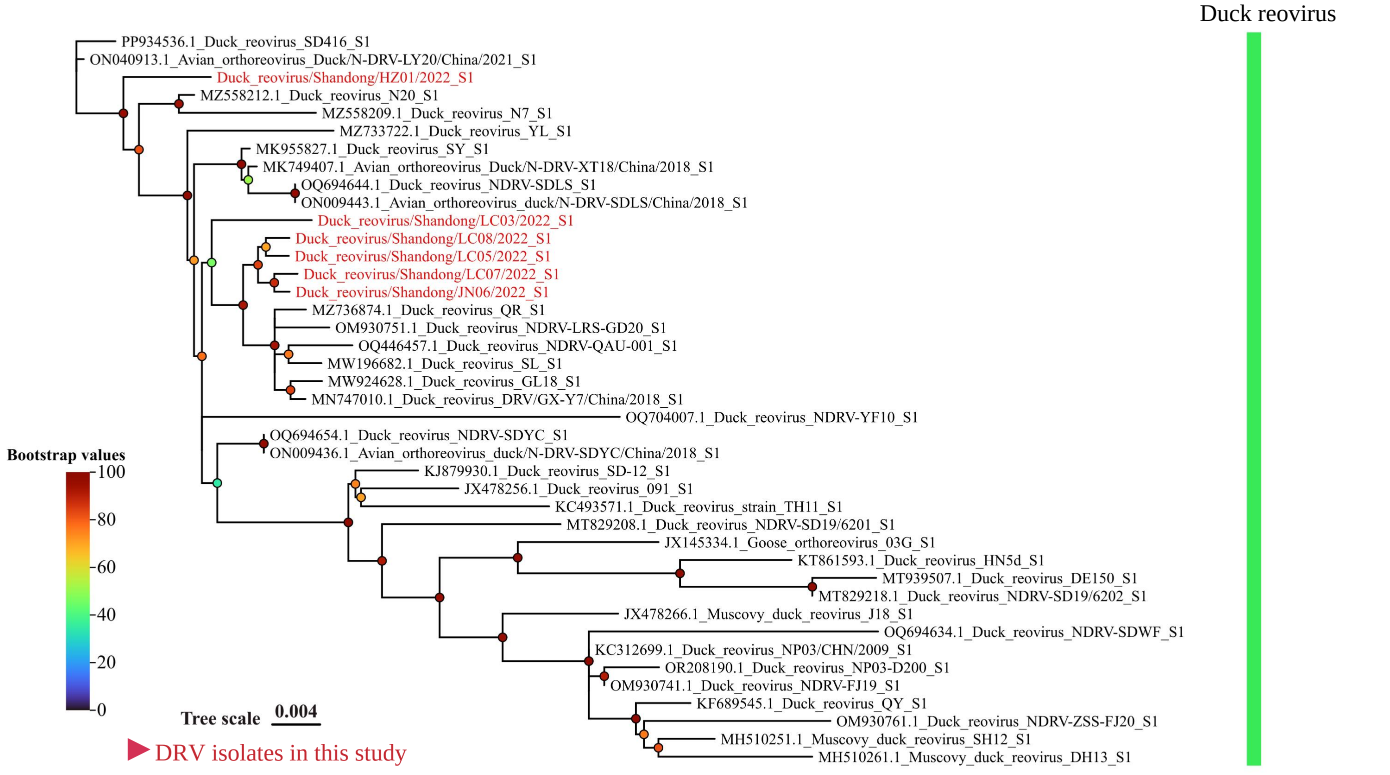

S2 phylogenetic tree

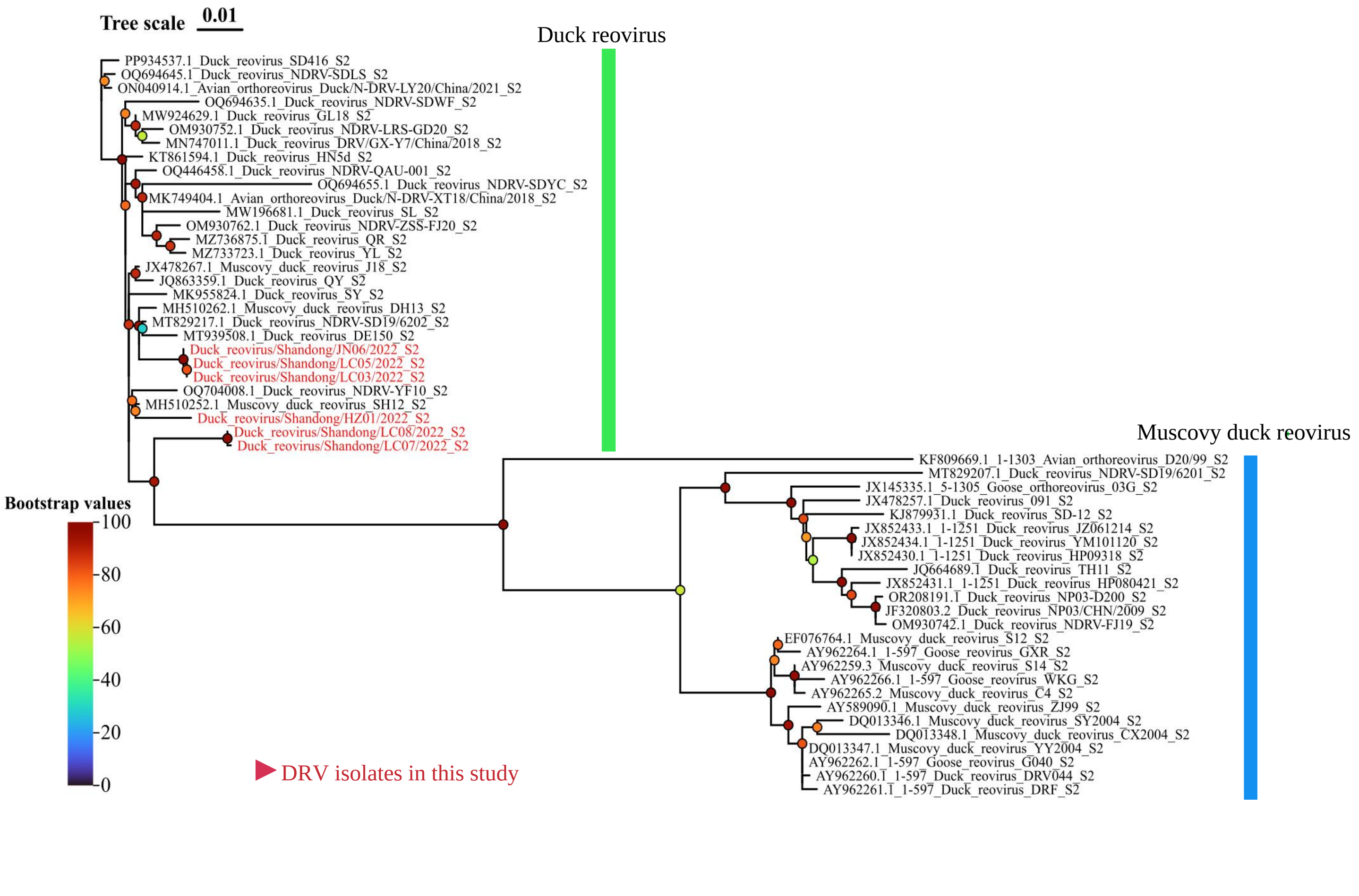

S3 phylogenetic tree

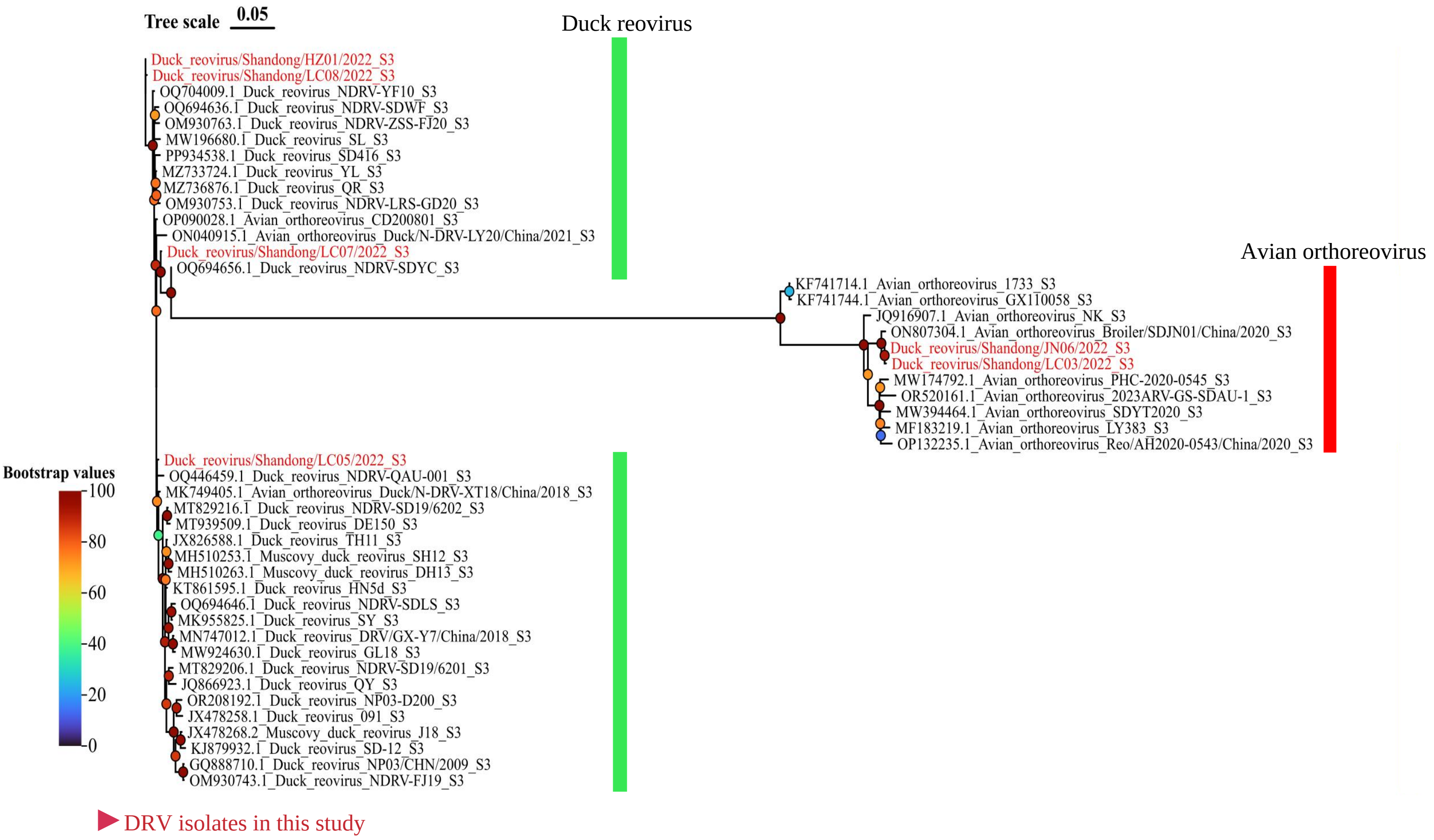

S4 phylogenetic tree

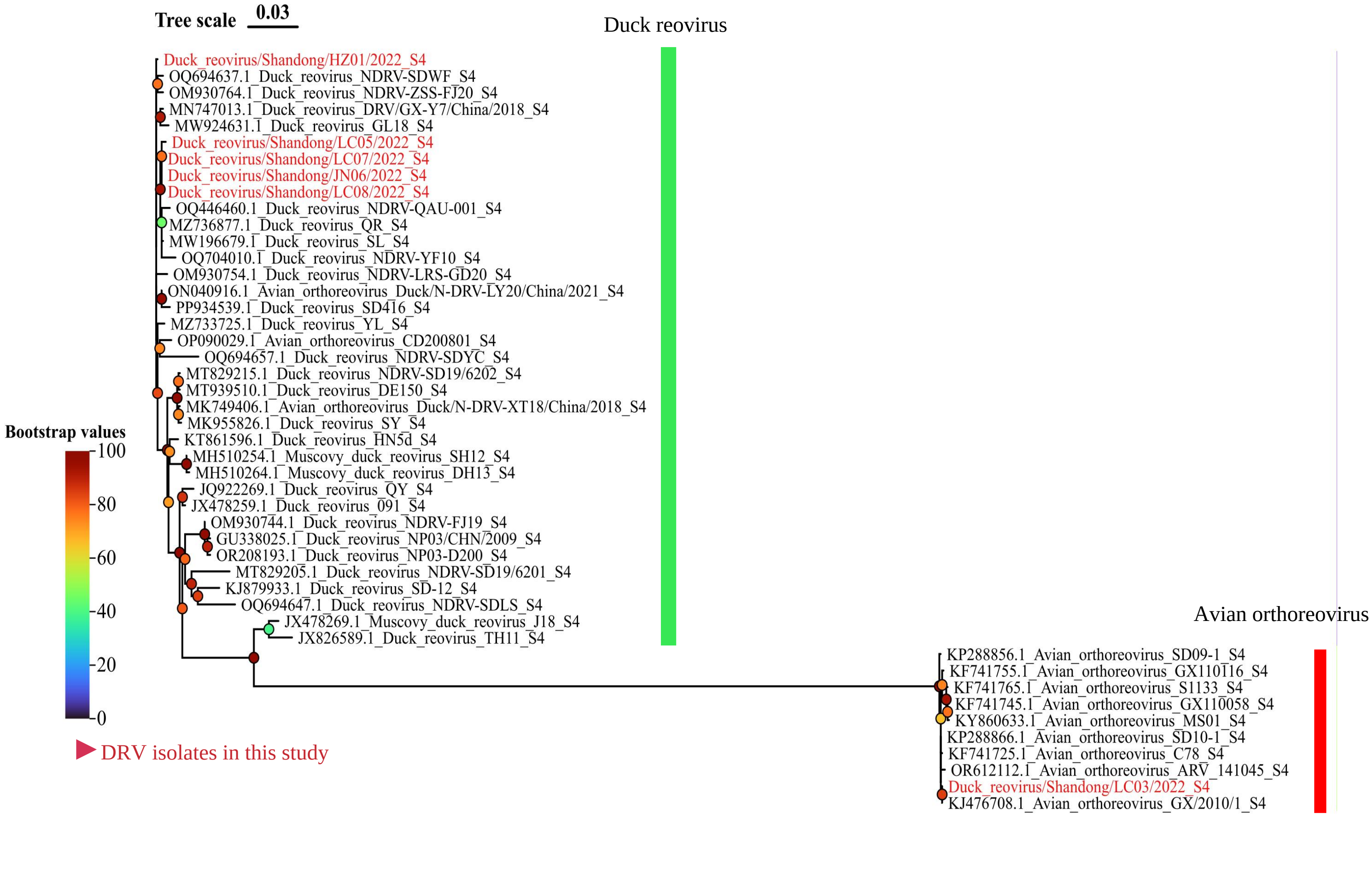

M1 phylogenetic tree

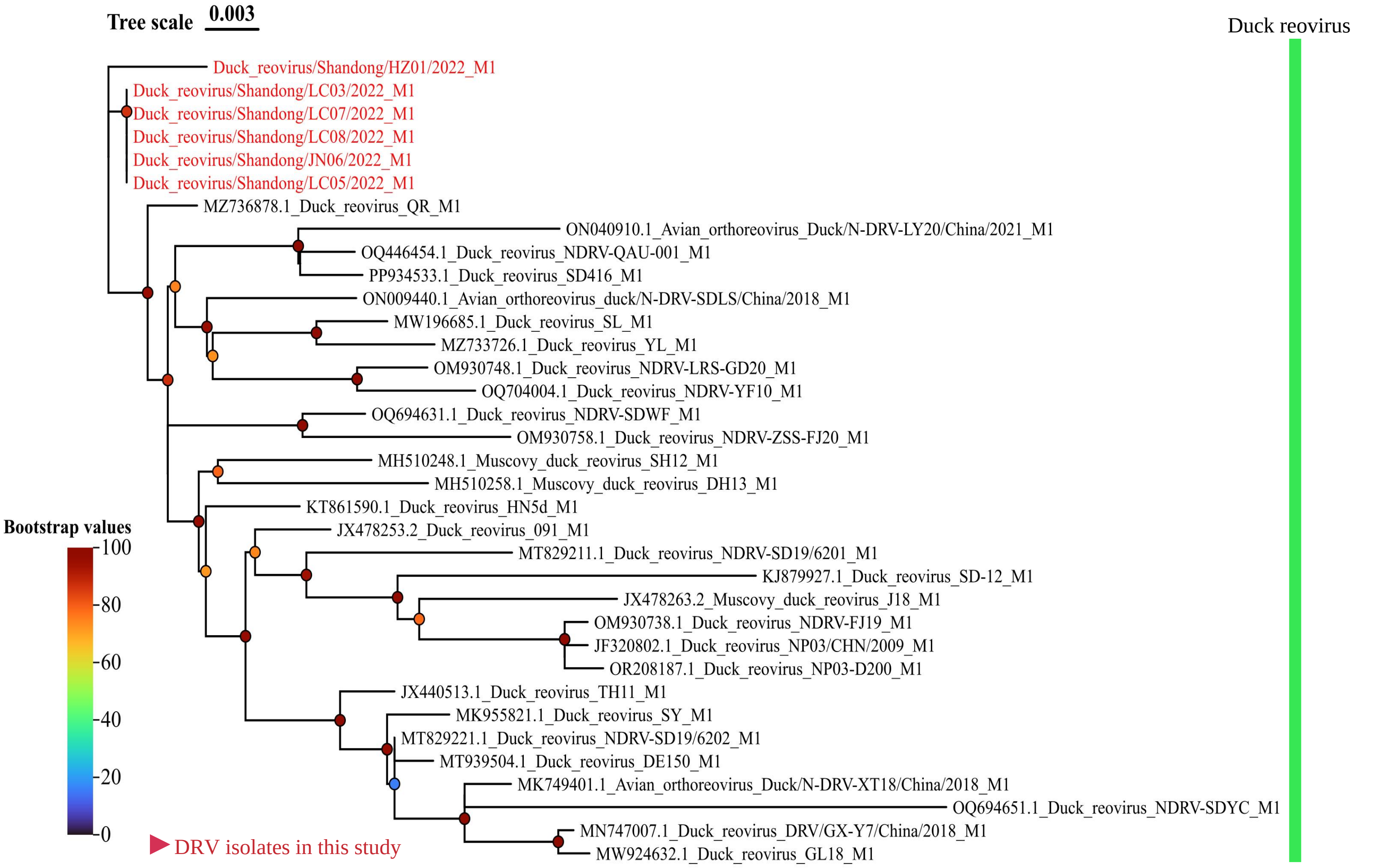

M2 phylogenetic tree

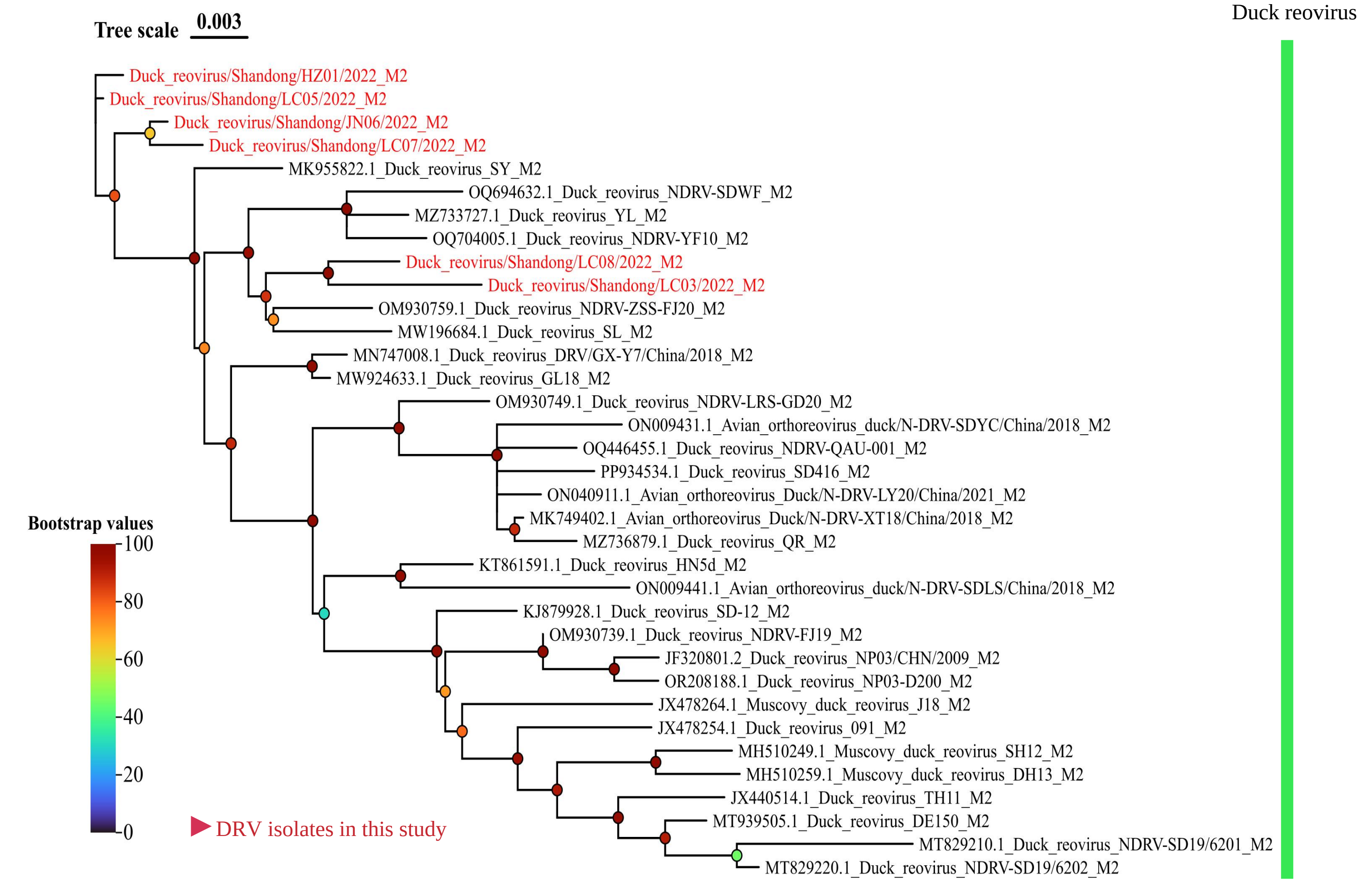

M3 phylogenetic tree

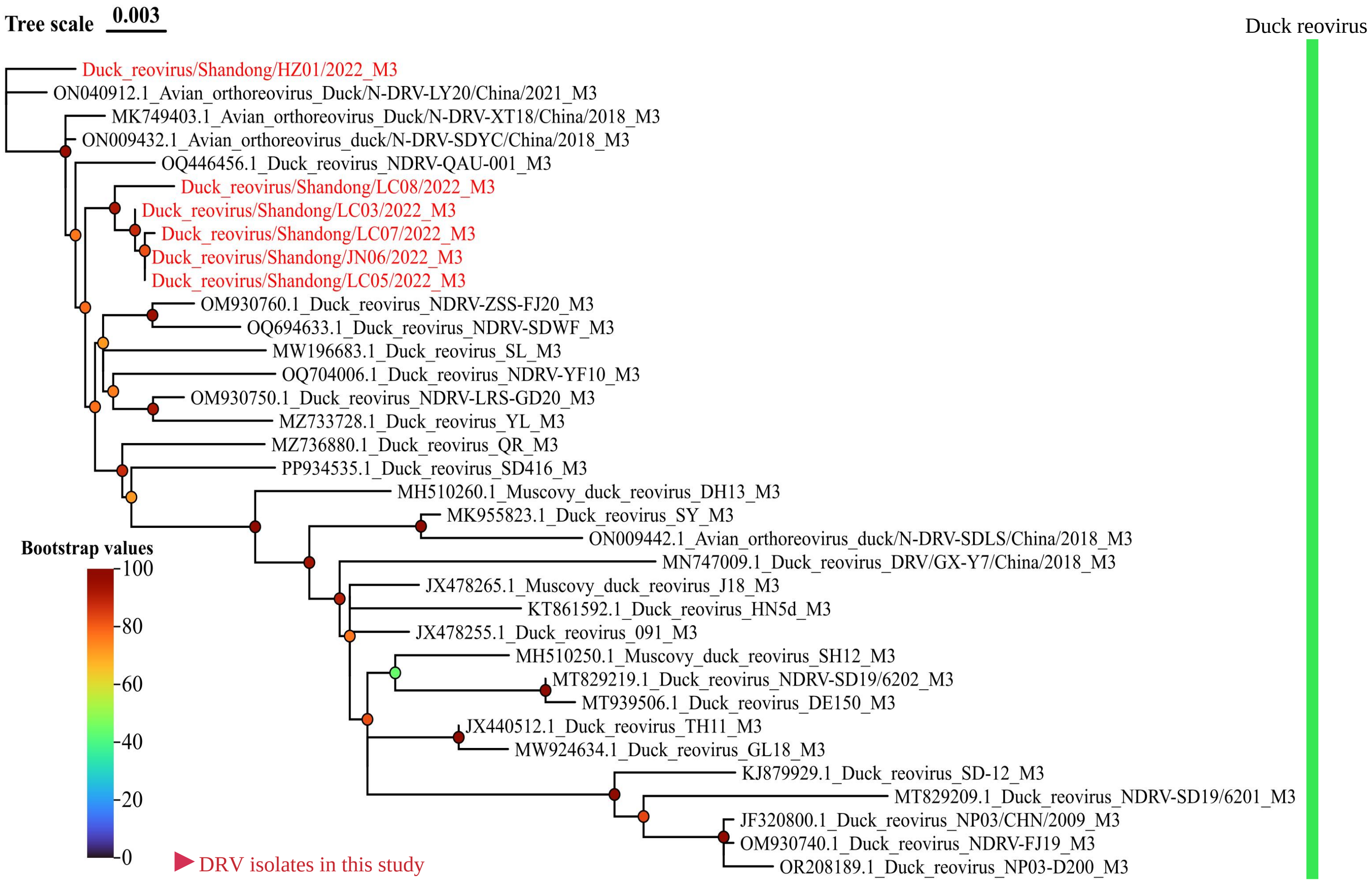

L1 phylogenetic tree

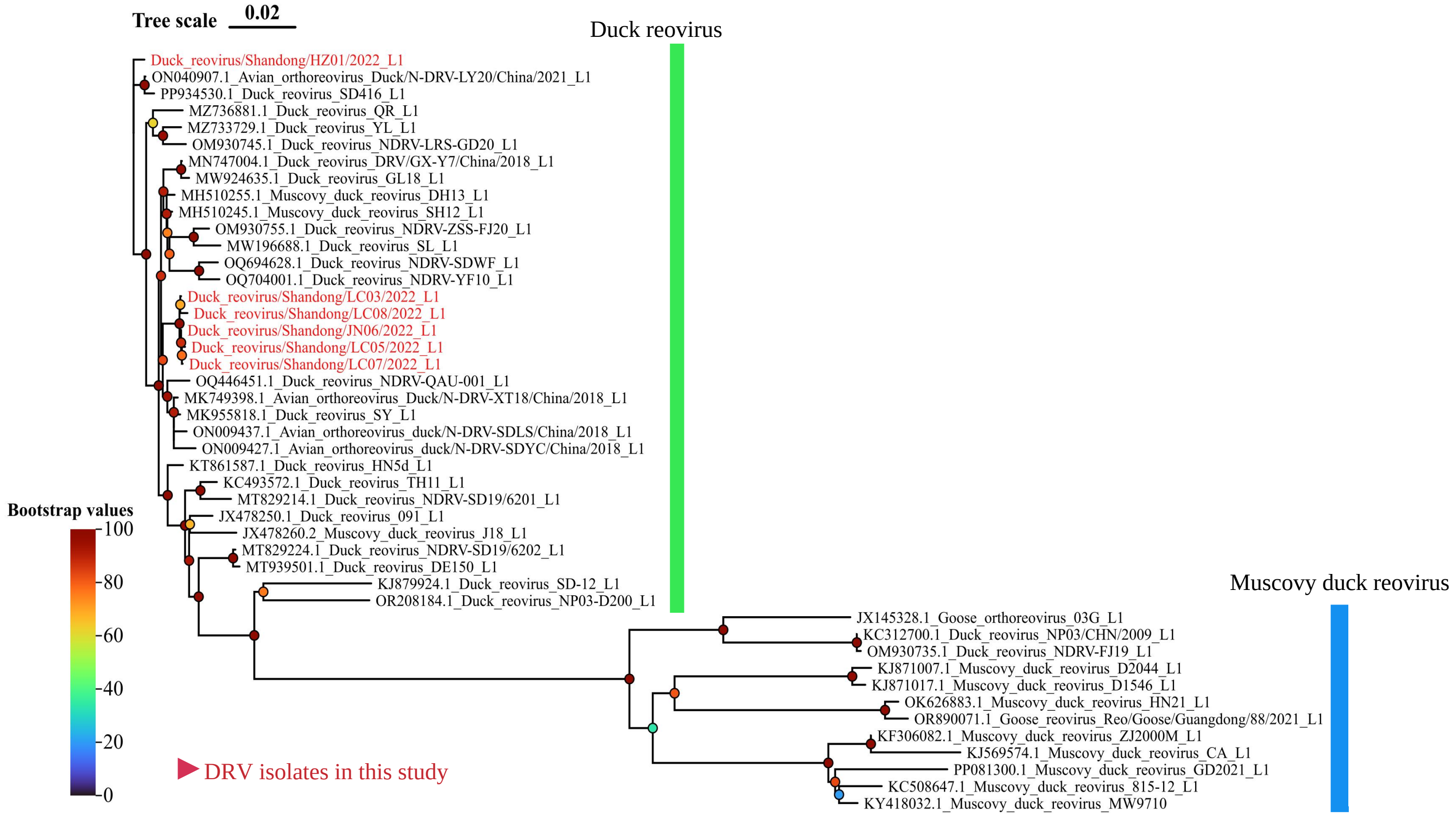

L2 phylogenetic tree

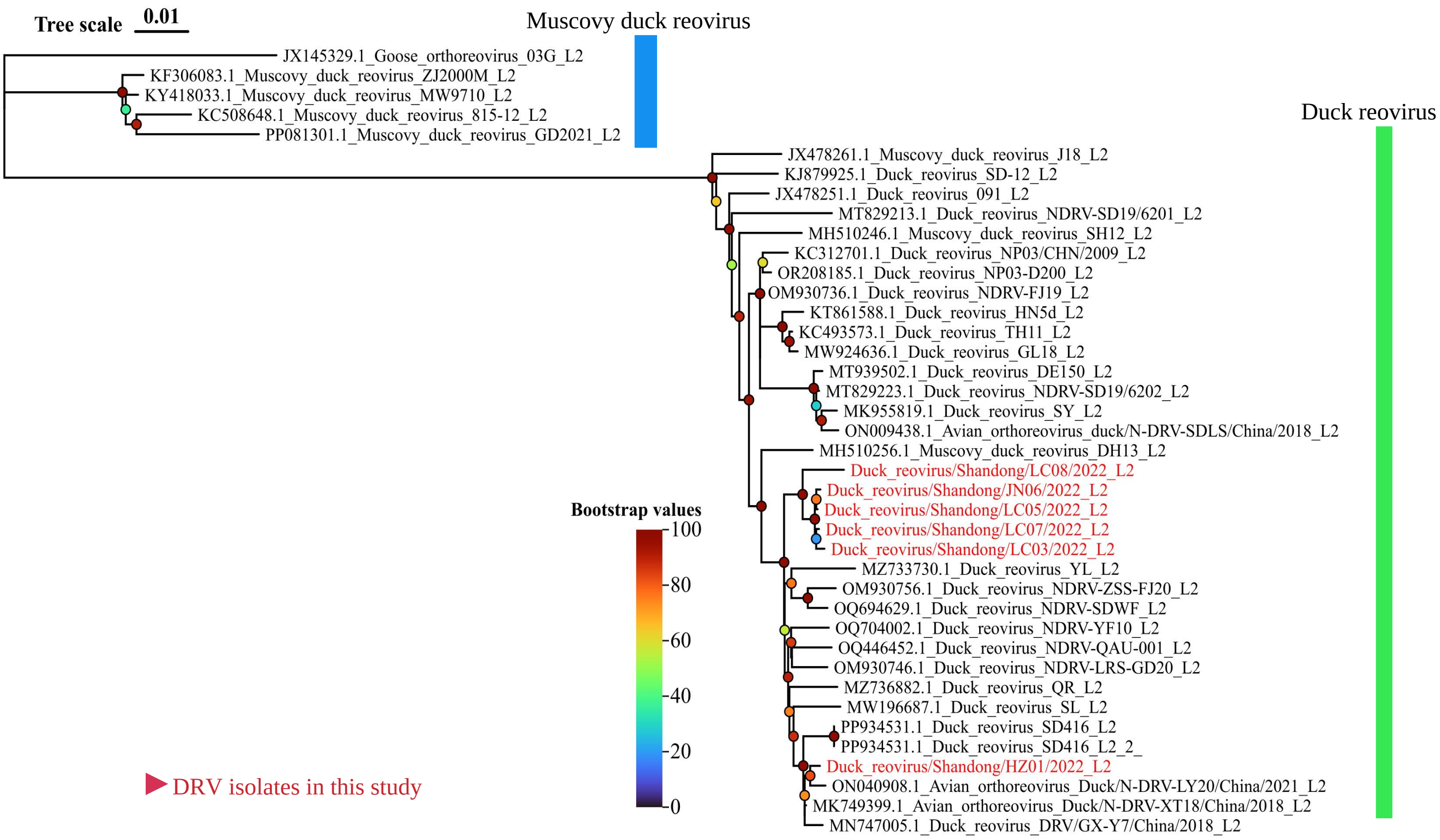

L3 phylogenetic tree

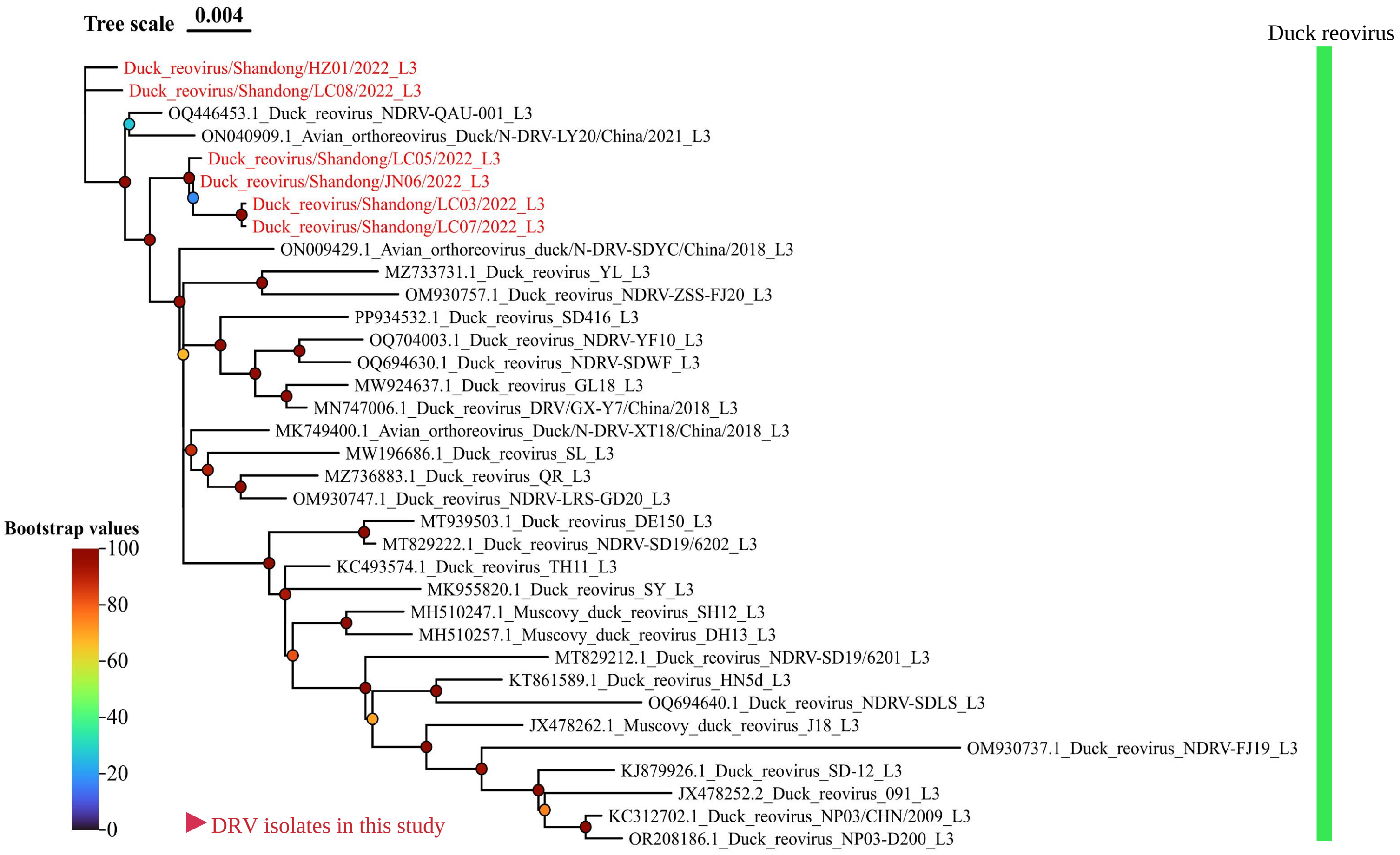

Supplement: Supplementary file 4 — Additional file 4. Phylogenetic trees of the genes (S1, S2, S3, S4, M1, M2, M3, L1, L2, and L3) of the duck reoviruses. The full-length sequences of each gene segment of the viruses were first aligned by MEGA 7.0, and a maximum likelihood (ML) phylogenetic tree was constructed via IQ-TREE with 1000 ultrafast bootstrap replicates. S1, 1568 nt; S2, 1251 nt (16-1266); S3, 1104 nt (31-1134); S4, 1104 nt (24-1127); M1, 2199 nt (14-2212); M2, 2028 nt (30-2057); M3, 1908 nt (25-1932); L1, 3882 nt (22-3903); L2, 3780 nt (15-3794); L3, 3858 nt (13-3870). [file 13567_2025_1470_MOESM4_ESM.pdf]
