# Supplementary material for: Genetics and biological characteristics of duck reoviruses isolated from ducks and geese in China
Source: Vet Res. 2025 Feb 6;56:30. doi: 10.1186/s13567-025-01470-7 (PMC11803967; doi:10.1186/s13567-025-01470-7)

Additional file 5. Pathological lesions in the bursa of Fabricius and the thymus of inoculated chickens

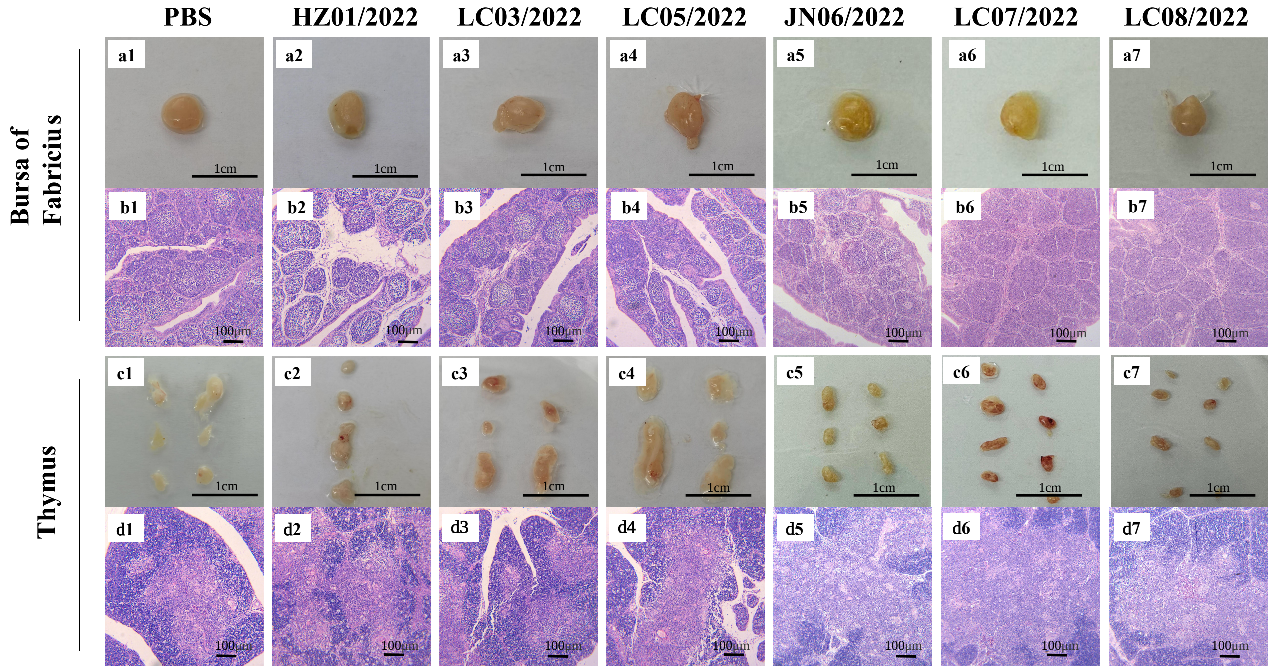

Supplement: Supplementary file 5 — Additional file 5. Pathological lesions in the bursa of Fabricius and the thymus of inoculated chickens. [file 13567_2025_1470_MOESM5_ESM.pdf]
